# Predominant NR1-positive puncta with limited PSD95 colocalization and close GluA2–EAAT2 apposition in the preBötzinger complex

**DOI:** 10.1007/s00418-026-02521-6

**Published:** 2026-08-06

**Authors:** Carol Alejandra Olmos-Pastoresa, Enrique Vázquez-Mendoza, Olivia Vázquez-Martínez, María Leonor López-Meraz, Luis Beltran-Parrazal, Consuelo Morgado-Valle

**Affiliations:** 1 Instituto de Investigaciones Cerebrales, Universidad Veracruzana, Dr. Castelazo Ayala S/N, 91190 Xalapa, Veracruz Mexico; 2 Instituto de Neurobiología, Universidad Nacional Autónoma de México, Boulevard Juriquilla 3001, 76230 Querétaro, Querétaro Mexico

**Keywords:** preBötzinger complex, AMPA receptor, NMDA receptor, EAAT2, Glutamate transport

## Abstract

The preBötzinger complex (preBötC) is a medullary network that generates the inspiratory phase of respiratory rhythm in mammals and depends critically on glutamatergic transmission. To characterize the anatomical organization of excitatory signaling elements within this network, we analyzed the distribution, morphology, and colocalization of the AMPA receptor subunit GluA2 and the NMDA receptor subunit NR1, together with the postsynaptic scaffold protein PSD95. We also used immunofluorescence and confocal microscopy to examine the spatial relationship between GluA2-positive puncta and the astrocytic glutamate transporter EAAT2 in the preBötC of adult male rats. NR1-positive puncta were more abundant and densely distributed than GluA2-positive puncta, whereas GluA2–NR1 colocalized puncta constituted only a small fraction of either receptor population. NR1-positive puncta also showed limited colocalization with PSD95-positive puncta, indicating that a substantial fraction of NR1 immunoreactivity is not associated with PSD95-defined postsynaptic domains under the present imaging conditions. In contrast, GluA2-positive puncta displayed a non-random spatial proximity with EAAT2 astrocytic profiles and were frequently located within submicron distances. Together, these findings reveal a spatially differentiated organization of glutamatergic elements in the preBötC, characterized by abundant non-PSD95-associated NR1-positive puncta and close apposition between GluA2-positive puncta and EAAT2-immunoreactive astrocytic profiles. This organization provides an anatomical framework for future studies testing how glutamate receptor localization and glutamate clearance mechanisms contribute to excitability and respiratory rhythm generation and modulation.

## Introduction

Breathing is a fundamental physiological process generated by neuronal circuits in the medulla. The preBötzinger complex (preBötC) is a medullary network responsible for generating the inspiratory rhythm and providing excitatory drive to downstream premotor and motor nuclei involved in breathing (Smith et al. 1991). Glutamatergic transmission within this network provides the principal excitatory drive required for rhythmogenesis (Koizumi et al. 2013).

Pharmacological studies have demonstrated that AMPA receptors (AMPARs) are essential for inspiratory burst generation. In neonatal medullary slices, AMPAR blockade suppresses inspiratory synaptic drive in preBötC neurons and progressively reduces inspiratory current amplitude until rhythmic activity ceases, thereby abolishing hypoglossal motor output (Smith et al. 1991; Funk et al. 1993; Ge and Feldman 1998; Morgado-Valle and Feldman 2007). In contrast, NMDA receptors (NMDARs) are not strictly required for baseline rhythm generation in vitro, as pharmacological blockade or genetic elimination does not disrupt rhythmic activity (Funk et al. 1993, 1997; Morgado-Valle and Feldman 2007). Nevertheless, NMDARs can sustain rhythm generation when AMPAR-mediated transmission is reduced, and Mg^2+^ blockade is relieved (Morgado-Valle and Feldman 2007), indicating that although AMPARs are necessary for rhythm generation, NMDARs contribute to the modulation of neuronal excitability and network dynamics.

Transcriptomic analyses have confirmed the expression of AMPAR (GluA1–4) and NMDAR (NR1/GluN1, NR2A–D, NR3A) subunits in the preBötC (Kallurkar et al. 2022). GluA2-containing AMPARs appear to predominate, consistent with Ca^2+^-impermeable receptor populations in this network (Paarmann et al. 2000; Kallurkar et al. 2022). The functional properties of NMDAR depend on subunit composition (Paoletti et al. 2013). However, beyond receptor subtype expression, their spatial organization within the preBötC remains poorly defined.

In other brain regions, glutamate receptors occupy synaptic, perisynaptic, and extrasynaptic domains (Newpher and Ehlers 2008), where they may respond to synaptic release, spillover, or ambient glutamate and engage distinct signaling pathways (Petralia 2012; Yao et al. 2022). Such spatial compartmentalization is increasingly recognized as a determinant of excitatory signaling, particularly in networks that rely on tonic drive (Kim et al. 2024). In the preBötC, extracellular glutamate levels are tightly regulated by astrocytic excitatory amino acid transporters (EAATs), including EAAT2/GLT-1 (Grass et al. 2004). Pharmacological inhibition of EAAT2 alters inspiratory rhythmic activity and modifies excitatory postsynaptic current kinetics (Morgado-Valle and Feldman 2004; Jørgensen et al. 2022). Both insufficient and excessive glutamate disrupt rhythm generation (Hülsmann et al. 2000; Morgado-Valle and Feldman 2004; Schnell et al. 2011), supporting the idea that preBötC function depends on an optimal extracellular glutamate tone.

Despite extensive functional studies, the anatomical organization of glutamatergic signaling components within the preBötC remains incompletely defined. In particular, the relative abundance and morphology of AMPAR and NMDAR-associated puncta, their association with postsynaptic scaffolding proteins, and their spatial relationship with astrocytic glutamate transporters have not been systematically examined.

Here, we mapped the distribution of GluA2, NR1, PSD95, and EAAT2 immunoreactivity in the adult rat preBötC using confocal microscopy and quantitative image analysis. Our goal was not to assign receptor populations definitively to synaptic, perisynaptic, or extrasynaptic membrane compartments, which would require ultrastructural or super-resolution approaches, but rather to determine whether NR1- and GluA2-positive puncta show distinct patterns of abundance, morphology, PSD95 association, and proximity to EAAT2-immunoreactive astrocytic profiles. This approach provides a first quantitative confocal map of receptor-associated and glutamate transporter-associated puncta within the preBötC, establishing an anatomical framework for generating testable hypotheses about how glutamate receptor-associated domains and glutamate clearance mechanisms may contribute to respiratory network excitability.

## Materials and methods

### Animals

Male Sprague–Dawley rats (3 months old;* n* = 9) were obtained from the animal facility of the Instituto de Neurobiología, Universidad Nacional Autónoma de México (Queretaro, Mexico). Animals were group-housed under controlled temperature (22 ± 2 °C) on a 12:12 h light/dark cycle with food and water ad libitum. All procedures complied with the *Guide for the Care and Use of Laboratory Animals* (NIH, 8th ed.) and NOM-062-ZOO-1999 and were approved by the Institutional Committee for the Care and Use of Laboratory Animals (CICUAL) of the Instituto de Investigaciones Cerebrales, Universidad Veracruzana (protocol 2021-004-a). Unless otherwise indicated, general laboratory reagents were obtained from Sigma-Aldrich (St. Louis, MO, USA).

### Tissue preparation

Rats were deeply anesthetized with a mixture of ketamine (75 mg/kg; Wildlife Pharmaceutical México, Mexico) and xylazine (5 mg/kg; Salud y Bienestar Animal, Mexico), administered intraperitoneally, and transcardially perfused with saline followed by 4% paraformaldehyde in 0.1 M phosphate buffer (PB). The brainstems were dissected, and a 5-mm coronal block containing the preBötC was obtained using anatomical landmarks. Tissue was post-fixed for 2 h and cryoprotected in 25% sucrose.

### Confocal microscopy

#### Immunofluorescence

Serial coronal sections (40 μm) were prepared on a cryostat (Leica CM1850 UV; Leica Biosystems, Nussloch, Germany) and stored in phosphate-buffered saline (PBS). Sections were collected in a 1:2 alternating series, with consecutive sections from each brainstem assigned to different immunofluorescence groups. After PBS rinses, sections underwent antigen retrieval in citrate buffer (pH 6.0), followed by quenching of endogenous peroxidase activity with 0.5% H_2_O_2_ and blocking of residual aldehydes with 0.05 M lysine in PBS. Sections were then incubated for 30 min in PBST (0.3% Triton X-100, 3% normal goat serum in PBS) to permeabilize tissue and block nonspecific binding.

Primary antibodies were mouse anti-GluA2 (1:400; Abcam Cat# ab192760, RRID: AB_3750166; Cambridge, UK), rabbit anti-NR1 (1:400; Abcam Cat# ab109182, RRID:AB_10862307; Cambridge, UK), rabbit anti-EAAT2 (1:500; Abcam Cat# ab41621, RRID:AB_941782; Cambridge, UK), and mouse anti-PSD95 (1:500; Abcam Cat# ab2723, RRID:AB_303248; Cambridge, UK). Sections were incubated for 48 h at 4 °C with the following combinations: GluA2–NR1, NR1–PSD95, and EAAT2–GluA2. Secondary antibodies were goat anti-mouse IgG Alexa Fluor 488 (1:500; Thermo Fisher Scientific Cat# A-11001, RRID: AB_2534069; Waltham, MA, USA) and goat anti-rabbit IgG Alexa Fluor 568 (1:750; Thermo Fisher Scientific Cat# A-11036, RRID:AB_10563566; Waltham, MA, USA). Sections were counterstained with DAPI (1:1000) and coverslipped with VECTASHIELD mounting medium (Cat. H-1000-10; Vector Laboratories, Newark, CA, USA).

**Fig. 1:**
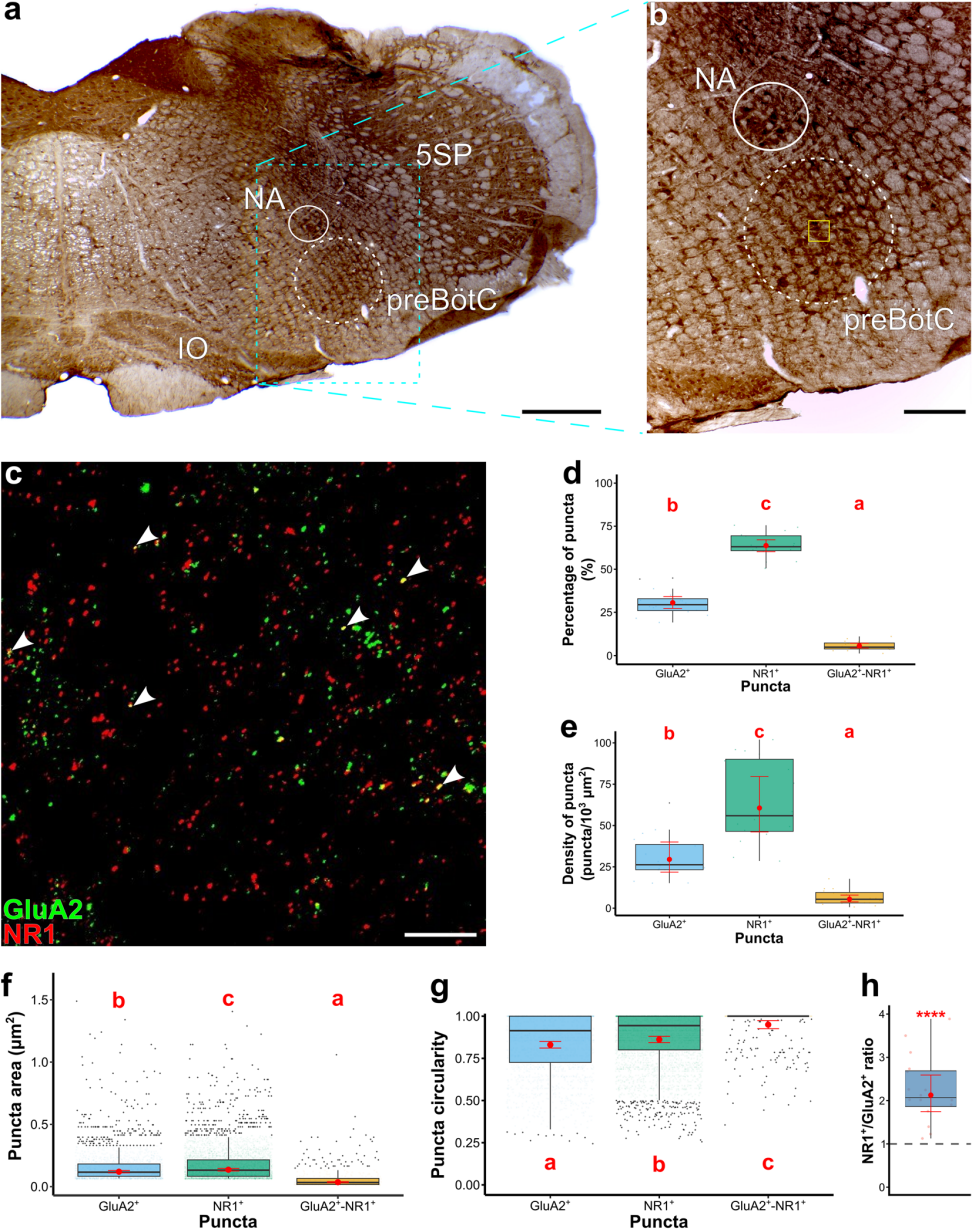
Distribution and morphological parameters of GluA2-positive, NR1-positive, and GluA2-positive–NR1-positive puncta in the preBötC. **a** Representative somatostatin-immunolabeled transverse medullary section showing the anatomical location of the preBötC. The dashed circle indicates the preBötC. The dashed rectangle denotes the region shown at higher magnification in **b**. **b** Higher magnification view of the preBötC region. The yellow square indicates the approximate area sampled for confocal imaging and quantitative analysis. **c** Representative confocal image showing GluA2-positive (green) and NR1-positive (red) puncta. Arrowheads indicate puncta exhibiting confocal-level overlap of GluA2 and NR1 immunoreactivity. Colocalization refers to confocal-level overlap of thresholded immunofluorescence signals and does not imply direct molecular interaction. Percentage (**d**), density (**e**), area (**f**), and circularity (**g**) of GluA2-positive, NR1-positive, and GluA2-positive–NR1-positive puncta. **h** Ratio of NR1-positive to GluA2-positive–NR1-positive puncta. GLMMs (images nested within animals) revealed significant effects of puncta type for all variables (all *p* < 0.0001). Boxplots show all observations; red symbols indicate back-transformed EMMs with 95% CI. Lowercase letters a–c denote statistically distinct groups: groups sharing a letter do not differ significantly, whereas different letters indicate significant pairwise differences (Sidak-adjusted, *p* < 0.0001). Asterisks indicate that the observed ratio differs significantly from 1.0 according to the GLMM analysis (****p < 0.0001).* n* = 16 images from four rats. Scale bars: **a** 500 μm; **b** 200 μm; **c** 10 μm. *IO* inferior olive, *NA* nucleus ambiguus, *preBötC* preBötzinger complex, *5SP* spinal trigeminal nucleus

For anatomical validation, somatostatin (SST) immunoreactivity was visualized using a guinea pig anti-SST antibody (1:500; Synaptic Systems Cat# 366 004, RRID:AB_2620126; Göttingen, Germany) followed by biotinylated goat anti-Guinea pig IgG secondary antibody (1:100; Abcam Cat# ab6907, RRID:AB_954847; Cambridge, UK), ABC amplification, and DAB-nickel detection (ABC kit; PK-6100; Vector Laboratories, Newark, CA, USA). SST immunoreactivity was used as an anatomical guide to identify the preBötC region within the ventrolateral medulla. Quantitative confocal fields were selected within the SST-defined preBötC region (Fig. 1a, b), avoiding neighboring regions of the ventral respiratory column whenever possible. ROI placement was based on anatomical landmarks and SST-positive neuronal distribution, not on the intensity or distribution of GluA2, NR1, PSD95, or EAAT2 fluorescence. Thus, SST labeling served only to support regional identification and was not included as a quantitative variable.

Antibody use was based on manufacturers’ validation data, published use in brain tissue, and the expected anatomical distribution of the corresponding antigens. All antibodies produced punctate or domain-like labeling patterns consistent with the known localization of glutamate receptor subunits, PSD95, EAAT2, and SST. Negative controls, including omission of primary antibodies, abolished the specific immunofluorescence signal, and single-label controls confirmed the absence of fluorescence bleed-through or cross-reactivity between channels under the acquisition settings used. Because genetic or peptide-blocking validation was not performed in the present tissue, the results are interpreted as patterns of GluA2-, NR1-, PSD95-, and EAAT2-immunoreactivity rather than as absolute receptor number, receptor surface expression, or definitive molecular localization.

Confocal images were acquired from the medial region of the preBötC (Fig. 1b) at comparable rostrocaudal levels across animals. All images within each immunolabeling group were acquired using identical laser, gain, pinhole, and detector settings to ensure comparability.

#### Image acquisition

Images were acquired using a Zeiss LSM 780 confocal microscope (Carl Zeiss Microscopy GmbH, Jena, Germany) with a ×63 Plan-Apochromat oil-immersion objective (NA 1.40, DIC M27). Four confocal z-stacks were collected from each brainstem at different coronal levels spanning the rostrocaudal extent of the SST-defined preBötC. The GluA2–NR1 and GluA2–EAAT2 datasets were obtained from four animals (16 images per dataset), whereas the NR1–PSD95 dataset was obtained from an independent cohort of five animals (20 images). Because the primary goal was to characterize receptor organization across the adult preBötC rather than to compare rostrocaudal subregions, confocal images were treated as repeated samples nested within animals in the statistical models. Images were acquired at 512 × 512 pixels with ×2 digital zoom. Z-step sizes were 0.43 μm for the GluA2–NR1 and NR1–PSD95 groups and 0.30 μm for the GluA2–EAAT2 group. Each image was averaged over eight scans with a scanning speed of 5 frames/s.

#### Image analysis

For this study, *puncta* (Fish et al. 2008) were defined as discrete segmented immunoreactive objects that satisfied the thresholding, area, and circularity criteria described below, whereas continuous labeling was defined as EAAT2-immunoreactive astrocytic profiles. Image processing and analysis were performed using ImageJ (v. 1.54p; NIH, USA). Images were initially separated into individual channels (*Split Channels*) and then deconvolved using the Iterative Deconvolve 3D plugin to improve signal clarity and reduce out-of-focus blur. Following deconvolution, brightness and contrast were adjusted to facilitate visualization during the segmentation process. These adjustments were used only to assist object identification and were not analyzed as quantitative variables.

For GluA2–NR1 and NR1–PSD95 analyses, green- and red-channel puncta were segmented separately from single-channel images. Threshold values were determined for each image by visual inspection using the ImageJ thresholding tool. Thresholds were selected to include discrete punctate immunoreactive structures while minimizing background fluorescence and were applied by the same observer throughout the study. Following threshold selection, puncta were identified using the *Analyze Particles* function. The same size and circularity parameters were applied to all puncta categories: an area range of 0.066–1.5 μm^2^ (corresponding to approximately 4–86 pixels at an image calibration of 0.132 μm/pixel) and circularity values from 0 to 1. These parameters were determined empirically to exclude background noise and minimize inclusion of overlapping or aggregated puncta that were unlikely to represent individual punctate structures.

Because threshold selection involved observer-guided segmentation, puncta identification should be regarded as an operational definition of immunoreactive objects rather than a direct measurement of receptor abundance or nanoscale receptor organization. Fluorescence intensity was not used as a quantitative outcome variable. Quantification was based on the number, area, circularity, and spatial relationships of segmented puncta.

Colocalization analysis was performed on merged images (*Merge Channels*) using the same thresholding criteria. Colocalized puncta were operationally defined as segmented objects showing spatial overlap of thresholded signals from the two fluorescence channels within the same pixel area, typically appearing as a yellow signal in merged images. Orthogonal projections (X, Y, Z planes) were examined using ZEISS ZEN 3.9 software to verify overlap in three dimensions and reduce false-positive colocalization due to axial displacement. Thus, colocalization in this study refers to confocal-level spatial overlap of immunofluorescence signals and does not imply molecular interaction or ultrastructural synaptic localization.

Puncta density was calculated as the number of puncta per 10^3^ μm^2^, and percentages were calculated relative to the total number of detected puncta per image. Because the NR1-GluA2 and NR1-PSD95 analyses were performed in independent immunolabeling series, absolute density values should not be interpreted as direct cross-experiment estimates of NR1 abundance.

Circularity was used strictly as a descriptive geometric parameter of segmented puncta shape, with values approaching 1 indicating more circular objects and lower values indicating more elongated or irregular objects. Because apparent object shape can be influenced by thresholding, signal-to-noise ratio, segmentation settings, the anisotropic confocal point-spread function, and the projection of three-dimensional structures into two-dimensional image representations, circularity was not interpreted as a measure of receptor-cluster geometry or as evidence of nanoscale receptor organization.

For GluA2–EAAT2 spatial analysis, the shortest edge-to-edge distances between GluA2-positive puncta and EAAT2-immunoreactive astrocytic profiles were measured using the DiAna plugin. Prior to analysis, images were processed by channel separation, manual adjustment of brightness and contrast, application of a Gaussian filter, and local contrast enhancement using CLAHE (with the fast option disabled to improve accuracy). Thresholds were applied to define objects, with size parameters of 3–100 pixels for GluA2 and 3–200,000 pixels for EAAT2. Distance measurements were computed using an edge-to-edge criterion.

Close proximity was operationally defined as ≤ 300 nm. This threshold was derived from the distribution of astrocyte–synapse distances reported by Octeau et al. (2018) and was selected as a biologically plausible estimate of astrocyte–synapse proximity. Given the resolution limits of confocal microscopy, this threshold was used as an operational measure of submicron spatial apposition.

### Statistical analysis

Percentage and ratio data were analyzed using beta generalized linear mixed-effects models (GLMMs); density using gamma GLMMs, and area and circularity using log-normal GLMMs. Puncta type was included as a fixed effect, with random intercepts for images nested within animals. GLMMs were fitted using the *glmmTMB* package.

Estimated marginal means (EMMs) were computed using the *emmeans* package, with Sidak-adjusted pairwise comparisons. Pairwise comparison results are presented using compact letter displays (CLDs), derived from the EMMs contrasts. Model diagnostics were assessed using the *DHARMa* package.

For the GluA2–EAAT2 spatial analysis, pooled distance distributions were compared with a uniform reference distribution bounded by the observed minimum and maximum distances using a Wilcoxon rank-sum test. To evaluate the robustness of the operational proximity criterion, sensitivity analyses were performed using distance thresholds of 100, 200, 300, 400, and 500 nm. For each threshold, the observed proportion of distances at or below the threshold was compared with the proportion expected under a uniform distribution spanning the observed distance range using chi-square goodness-of-fit tests. This analysis was used as an exploratory test of non-random proximity; it does not model the full spatial geometry of the tissue, object density, or EAAT2 domain occupancy.

Analyses were conducted in R (v4.5.0). Results are reported as EMMs with 95% confidence intervals (CI). Statistical significance was set at *p* ≤ 0.05.

## Results

### Differential distribution of GluA2-positive and NR1-positive puncta in the preBötC

In the NR1–GluA2 group, 7079 puncta were identified (*n* = 16 images from four rats; Fig. 1c). Puncta type significantly affected relative abundance (*χ*^2^(2) = 98.29, *p* < 0.0001). NR1-positive puncta were the most abundant (63.7%, 95% CI 59.4–67.8), exceeding both GluA2-positive puncta (30.6%, 95% CI 26.6–34.9) and GluA2-positive–NR1-positive colocalized puncta (5.7%, 95% CI 4.3–7.6; all *p* < 0.0001; Fig. 1d). Although colocalized puncta were uncommon, this result indicates that GluA2-positive and NR1-positive puncta largely occupy non-overlapping confocal domains under the present segmentation criteria.

Puncta density also differed significantly (*χ*^2^(2) = 69.64, *p* < 0.0001). NR1-positive puncta showed the highest density (60.6 puncta/10^3^ μm^2^, 95% CI 43.5–84.5), followed by GluA2-positive (29.5 puncta/10^3^ μm^2^, 95% CI 20.4–42.8), whereas GluA2-positive–NR1-positive colocalized puncta had the lowest density (5.5 puncta/10^3^ μm^2^, 95% CI 3.5–8.6; all *p* < 0.0001; Fig. 1e).

Morphometric analysis revealed significant differences in both area (*χ*^2^(2) = 549.16, p < 0.0001) and circularity (*χ*^2^(2) = 206.8, *p* < 0.0001). NR1-positive puncta exhibited the largest mean area (0.136 μm^2^, 95% CI 0.126–0.147), followed by GluA2-positive puncta (0.120 μm^2^, 95% CI 0.111–0.130), whereas colocalized puncta were markedly smaller (0.036 μm^2^, 95% CI 0.032–0.040; Fig. 1f). Circularity was highest in colocalized puncta (0.950, 95% CI 0.926–0.974), followed by NR1-positive puncta (0.862, 95% CI 0.843–0.881) and GluA2-positive puncta (0.830, 95% CI 0.811–0.850; all pairwise *p* < 0.0001; Fig. 1g).

The NR1-positive/GluA2-positive ratio was significantly greater than 1 (2.13, 95% CI 1.75–2.58; *p* < 0.0001), whereas the colocalization ratios relative to NR1-positive puncta (0.08, 95% CI 0.05–0.13) and GluA2-positive puncta (0.17, 95% CI 0.11–0.26) were significantly below 1 (*p* < 0.0001; Fig. 1h), indicating that GluA2-positive–NR1-positive colocalized puncta constituted only a small fraction of either population.

Together, these findings indicate that NR1-positive puncta are more abundant than GluA2-positive puncta, whereas GluA2–NR1 colocalized puncta constitute only a small fraction of either population. Thus, most NR1-positive puncta in the adult rat preBötC do not overlap with GluA2-positive puncta at confocal resolution.

### Limited colocalization of NR1-positive puncta with PSD95-positive domains in the preBötC

**Fig. 2:**
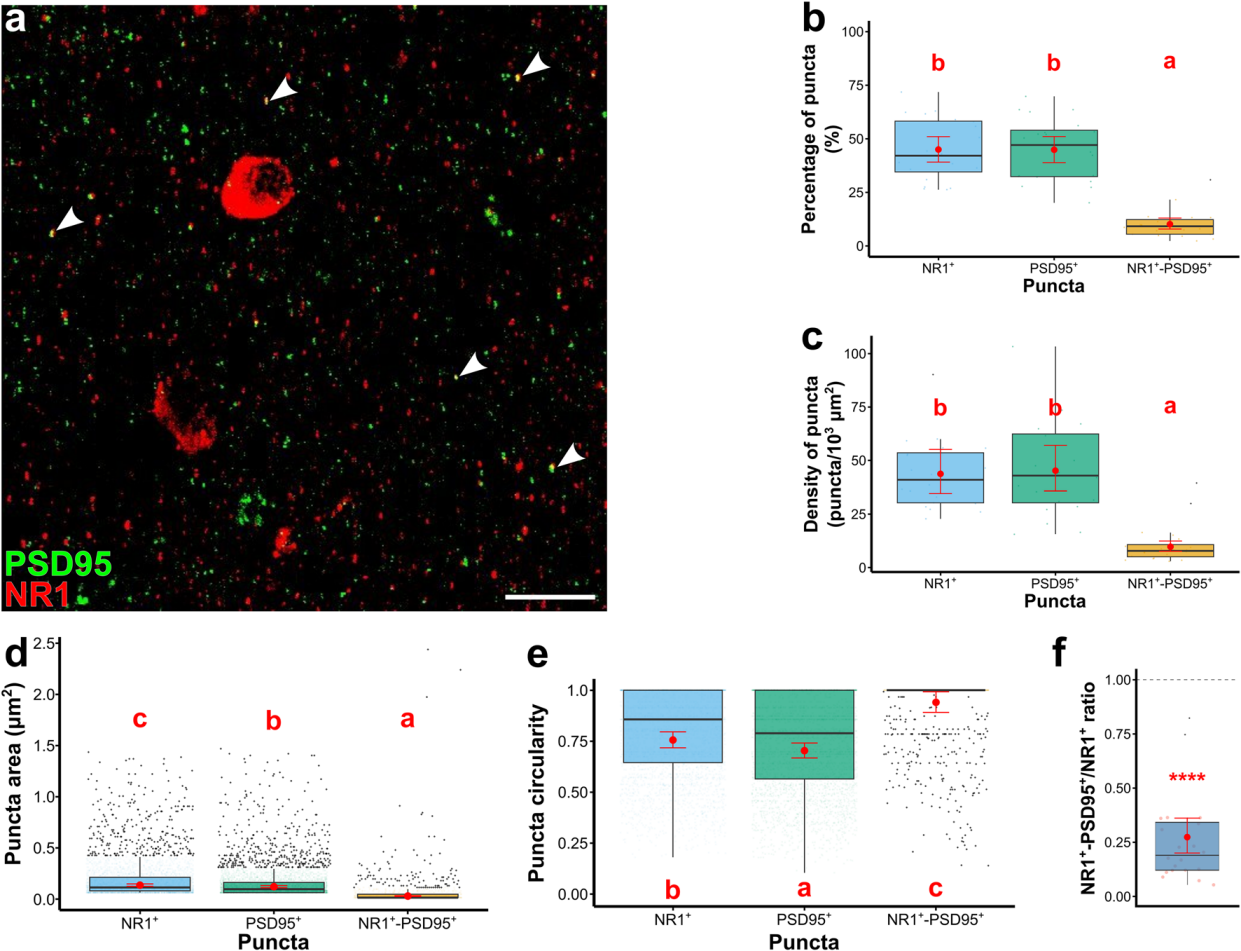
Distribution and morphological characteristics of NR1-positive, PSD95-positive, and NR1-positive–PSD95-positive puncta in the preBötC. **a** Representative confocal image showing NR1-positive (red) and PSD95-positive (green) puncta. Arrowheads indicate puncta exhibiting confocal-level overlap of NR1 and PSD95 immunoreactivity. The limited overlap between NR1-positive and PSD95-positive puncta is interpreted as reduced association with PSD95-defined domains and should not be considered definitive ultrastructural evidence of extrasynaptic localization. Percentage (**b**), density (**c**), area (**d**), and circularity (**e**) of NR1-positive, PSD95-positive, and NR1-positive–PSD95-positive puncta. **f** Ratio of NR1-positive–PSD95-positive to NR1-positive puncta. GLMMs (images nested within animals) revealed significant effects of puncta type for all variables (all* p* < 0.0001). Boxplots show all observations; red symbols denote back-transformed EMMs with 95% CI. Lowercase letters a–c denote statistically distinct groups: groups sharing a letter do not differ significantly, whereas different letters indicate significant pairwise differences (Sidak-adjusted, *p* < 0.0001). Asterisks indicate that the observed ratio differs significantly from 1.0 according to the GLMM analysis (****p < 0.0001).* n* = 20 images from five rats. Scale bar: 10 μm

In the NR1–PSD95 group, 8652 puncta were quantified (*n* = 20 images from five rats; Fig. 2a). Puncta type significantly affected percentage (*χ*^2^(2) = 48.42, *p* < 0.0001). NR1-positive puncta (45.0%, 95% CI 37.9–52.3) and PSD95-positive puncta (44.9%, 95% CI 37.6–52.4) were present in comparable proportions (*p* = 1), and both exceeded the proportion of NR1-positive–PSD95-positive colocalized puncta (10.2%, 95% CI 7.5–13.8; *p* < 0.0001; Fig. 2b).

Densities mirrored these proportions (*χ*^2^(2) = 56.31, *p* < 0.0001): NR1-positive puncta (43.71 puncta/10^3^ μm^2^, 95% CI 32.94–58.0) and PSD95-positive puncta (45.20 puncta/10^3^ μm^2^, 95% CI 34.04–60.0) were similar (*p* = 0.994) and both exceeded the density of colocalized puncta (9.77 puncta/10^3^ μm^2^, 95% CI 7.28–13.1; *p* < 0.0001; Fig. 2c).

Area (*χ*^2^(2) = 1143.9, *p* < 0.0001) and circularity (*χ*^2^(2) = 551.85, *p* < 0.0001) also differed significantly. NR1-positive puncta had the largest mean area (0.138 μm^2^, 95% CI 0.127–0.150), PSD95-positive puncta were intermediate in size (0.123 μm^2^, 95% CI 0.113–0.134), and colocalized puncta were the smallest (0.032 μm^2^, 95% CI 0.029–0.036; Fig. 2d). Circularity was highest in colocalized puncta (0.943, 95% CI 0.889–1.000; all pairwise *p* < 0.0001; Fig. 2e).

The NR1-positive–PSD95-positive/NR1-positive ratio was 0.274 (95% CI 0.201–0.361; *p* < 0.0001; Fig. 2f), indicating that the majority of NR1-positive puncta (approx. 1:3.7) lack PSD95 colocalization. Together, these findings indicate that a large fraction of NR1-positive puncta do not overlap with PSD95-positive puncta at confocal resolution, consistent with a prominent non-PSD95-associated NR1-positive population in the preBötC.

### Non-random spatial association between GluA2-positive puncta and EAAT2-immunoreactive astrocytic profiles in the preBötC

Having found that a large proportion of NR1-positive puncta did not overlap with PSD95-positive domains, we next asked whether GluA2-positive puncta showed a spatial relationship with EAAT2-immunoreactive astrocytic domains. Because EAAT2 plays a central role in shaping extracellular glutamate levels, we examined the spatial proximity between GluA2-positive puncta and EAAT2-positive domains to explore whether AMPAR-associated puncta are anatomically positioned near glutamate clearance machinery.

**Fig. 3:**
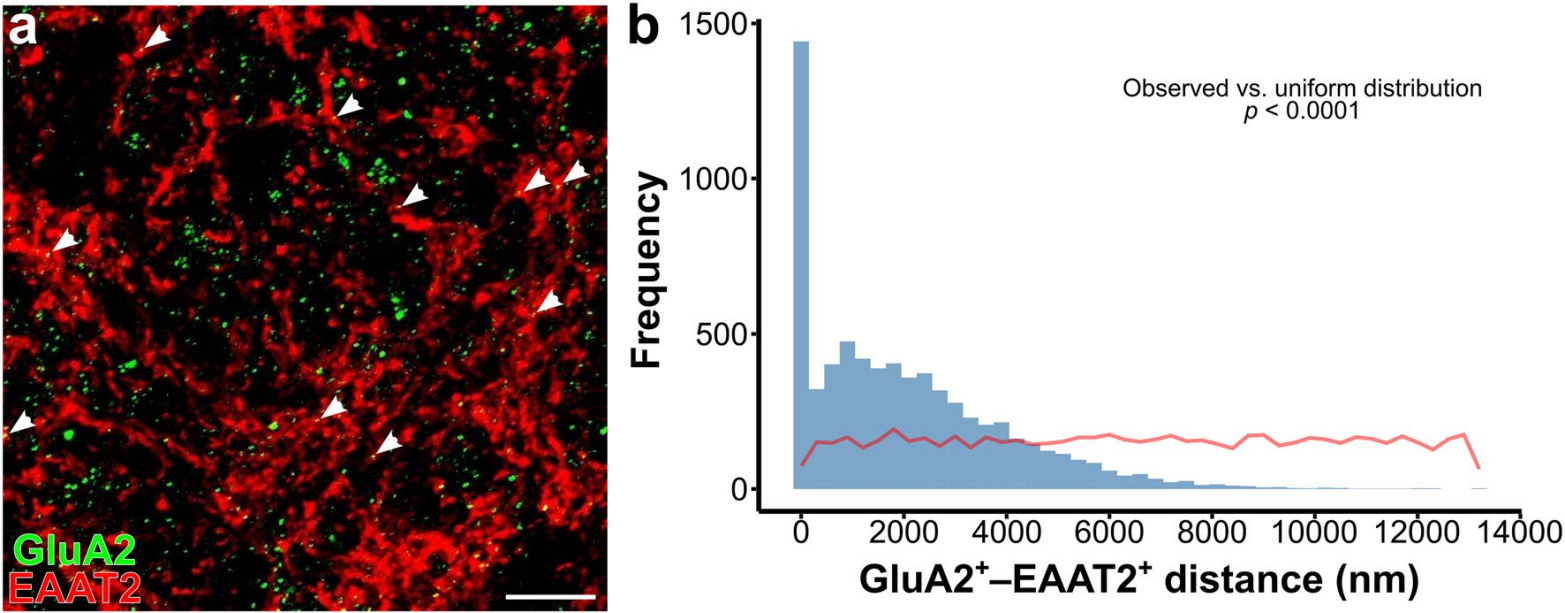
Enrichment of GluA2-positive puncta within the operational submicron proximity range of EAAT2-positive domains. **a** Representative confocal image showing GluA2-positive puncta (green) and EAAT2-positive domains (red). Arrowheads indicate representative GluA2-positive puncta exhibiting overlap or immediate apposition with EAAT2-positive domains. **b** Histogram of shortest edge-to-edge distances between GluA2-positive puncta and the nearest EAAT2-positive domain (300-nm bins), based on 6869 nearest-neighbor distances measured from 16 images obtained from four rats. The red line represents a simulated uniform distribution spanning the minimum (0 nm) and maximum (13,171 nm) values of the observed distance distribution. The observed distribution differed significantly from the uniform distribution (*p* < 0.0001), indicating a non-random enrichment of GluA2-positive puncta at short distances from EAAT2-positive domains. Scale bar: 10 μm

In the GluA2–EAAT2 group, 6869 nearest-neighbor distances were measured (*n* = 16 images from four rats; Fig. 3a). Distances ranged from 0 to 13,171 nm (Q1 387 nm; median 1632 nm; Q3 3118 nm). Notably, 1400 distances (20.4% of all measurements) were equal to 0 nm, indicating overlap of segmented GluA2-positive puncta and EAAT2-immunoreactive domains according to the edge-to-edge distance criterion.

The observed distribution differed from a simulated uniform distribution (Wilcoxon rank-sum test: *W* = 7,330,762, *p* < 0.0001; Fig. 3b), indicating that GluA2-positive puncta were not randomly distributed with respect to EAAT2-positive domains under this exploratory distance-based comparison.

To further assess close spatial associations, we examined the proportion of GluA2-positive puncta located within a series of operational proximity thresholds ranging from 100 to 500 nm. Across all thresholds tested, the observed proportion of puncta located near EAAT2-immunoreactive domains was substantially greater than expected under a uniform distance distribution (Table 1). At the predefined 300 nm threshold, 23.5% of measured distances fell within this range, compared with 2.3% expected under the null distribution, representing a 10.3-fold enrichment (*χ*^2^(1) = 13,952.08, *p* < 0.0001). Similar enrichments were observed at 100, 200, 400, and 500 nm thresholds, indicating that the observed spatial association between GluA2-positive puncta and EAAT2-positive astrocytic domains was robust to the choice of proximity criterion.

**Table 1:** Sensitivity analysis of GluA2–EAAT2 spatial proximity using different operational distance thresholds. Expected values were calculated assuming a uniform distribution of distances between 0 and the maximum observed distance

Threshold (nm)	Observed (%)	Expected (%)	Fold enrichment	*p* value
100	20.38	0.76	26.84	< 0.0001
200	21.59	1.52	14.22	< 0.0001
300	23.54	2.28	10.34	< 0.0001
400	25.13	3.04	8.27	< 0.0001
500	26.55	3.80	6.99	< 0.0001

## Discussion

This study provides a quantitative confocal analysis of GluA2, NR1, PSD95, and EAAT2 immunoreactivity in the adult rat preBötC. Three main findings emerged. First, NR1-positive puncta were more abundant and densely distributed than GluA2-positive puncta. Second, NR1-positive puncta showed limited overlap with both GluA2-positive and PSD95-positive puncta, indicating that a substantial fraction of NR1 immunoreactivity is not associated with GluA2- or PSD95-defined domains under the present imaging conditions. Third, GluA2-positive puncta were frequently located in close spatial proximity to EAAT2-positive astrocytic domains.

Together, these findings support a spatially differentiated organization of glutamatergic elements in the preBötC and identify anatomical features that may be relevant to glutamate signaling within respiratory networks. In particular, the presence of a prominent non-PSD95-associated NR1-positive population and the close association between GluA2-positive puncta and EAAT2-positive astrocytic domains provide an anatomical framework for investigating how receptor organization and glutamate clearance mechanisms contribute to respiratory network function.

### Neuroanatomical characterization of AMPA and NMDA receptors in the preBötC

NR1-positive puncta in the adult rat preBötC were approximately twice as abundant as GluA2-positive puncta. This is notable because AMPAR-mediated transmission is essential for inspiratory burst generation, whereas NMDARs are not strictly required for baseline rhythm generation under standard in vitro conditions (Smith et al. 1991; Funk et al. 1993; Morgado-Valle and Feldman 2007). The abundance of NR1 immunoreactivity suggests that NMDAR-associated signaling elements may be relevant to modulatory or state-dependent aspects of preBötC excitability. This interpretation is broadly consistent with physiological evidence showing that NMDARs can sustain rhythmic activity under conditions in which AMPAR-mediated transmission is reduced and Mg^2+^ block is relieved (Morgado-Valle and Feldman 2007).

Our findings provide an anatomical context for these physiological observations by demonstrating abundant NR1 immunoreactivity, much of which was not associated with GluA2-positive puncta at confocal resolution. To our knowledge, the relative abundance and spatial association of AMPAR- and NMDAR-associated puncta have not previously been quantified in the adult preBötC. Because glutamate receptor expression undergoes developmental changes (Liu and Wong-Riley 2002, 2010), and many functional studies of the preBötC have been performed in neonatal preparations, future studies will be required to determine whether similar anatomical relationships are preserved across developmental stages.

### Association of NR1-positive puncta with GluA2- and PSD95-defined domains

Most NR1-positive puncta did not overlap with GluA2-positive puncta and showed limited colocalization with PSD95, indicating a prominent non-PSD95-associated NR1-positive population at confocal resolution. In other brainstem regions, such as the vestibular nuclei, NR1 immunoreactivity has been reported to be co-expressed with AMPAR subunits within the same neurons, although the extent of co-expression varies among receptor subtypes and vestibular nuclei (Chen et al. 2000). Importantly, that study evaluated receptor co-expression at the level of neuronal somata and dendrites rather than the spatial relationship between individual puncta. Consequently, neuronal co-expression of NMDAR and AMPAR subunits does not necessarily predict extensive puncta-level colocalization. The limited NR1-GluA2 overlap observed in the present study may therefore reflect a distinct subcellular organization of glutamate receptor-associated domains in the preBötC rather than a discrepancy with previous reports. Future studies using object-based super-resolution imaging or ultrastructural approaches will be required to determine whether the spatial relationships between AMPAR- and NMDAR-associated domains differ across brainstem regions.

Glutamate receptors can occupy synaptic, perisynaptic, extrasynaptic, and intracellular compartments, and receptor localization influences the glutamatergic signals to which they are exposed (Newpher and Ehlers 2008; Petralia 2012; Papouin and Oliet 2014). The limited overlap between NR1 and PSD95 observed here indicates that a substantial fraction of NR1 immunoreactivity is located outside PSD95-defined domains. Although PSD95 is a widely used marker of excitatory postsynaptic specializations (Petralia 2012), NMDARs can also associate with alternative scaffold proteins and signaling complexes outside PSD95-enriched domains (Allison et al. 1998; Petralia et al. 2010). Therefore, the absence of PSD95 colocalization does not permit assignment of NR1-positive puncta to specific subcellular compartments. Nevertheless, the abundance of this non-PSD95-associated NR1-positive population identifies a previously unquantified anatomical feature of the adult preBötC that warrants further investigation.

### Puncta morphology and receptor organization

Puncta area and circularity differed among GluA2-positive, NR1-positive, and colocalized puncta, indicating that the segmented immunoreactive objects were not morphologically identical across markers. Colocalized puncta were smaller and more circular than single-label puncta. Because puncta morphology measured by confocal microscopy is influenced by optical resolution and anisotropy, thresholding, antibody labeling density, and segmentation parameters, these measurements should be interpreted as descriptive morphometric features rather than as direct evidence of receptor-cluster geometry or nanoscale receptor architecture. Determining their structural or functional significance will require higher-resolution approaches.

### Spatial proximity between GluA2-positive puncta and EAAT2-positive domains in the preBötC

GluA2-positive puncta were non-randomly enriched near EAAT2-positive astrocytic domains within a submicron range. Because EAAT2 is the major astrocytic glutamate transporter in many brain regions and is expressed within respiratory networks, this arrangement is consistent with an anatomical organization that could influence glutamate diffusion and clearance near excitatory signaling elements (Hama et al. 2004; Chai et al. 2017; Khakh and Sofroniew 2015; Allen and Eroglu 2017). The most conservative conclusion is that GluA2-positive puncta are anatomically positioned near EAAT2-immunoreactive domains. Methodological limitations of this proximity analysis are discussed in the “Caveats” section.

### Working anatomical hypothesis

The present findings support a working anatomic hypothesis in which a non-PSD95-associated NR1 immunoreactive population and EAAT2-positive astrocytic domains represent distinct organizational features of glutamatergic signaling in the preBötC. Although the functional significance of these arrangements cannot be determined from the present data, they identify anatomical patterns potentially relevant to extracellular glutamate regulation and neuronal excitability.

These observations generate testable hypotheses regarding glutamatergic organization in the adult preBötC that will require validation using cell-type-specific, ultrastructural, and functional approaches.

### Caveats

Several methodological considerations should be noted.

First, confocal microscopy does not provide sufficient spatial resolution to assign receptor puncta to specific membrane compartments. Therefore, the non-PSD95-associated NR1-positive population identified here cannot be classified as synaptic, perisynaptic, or extrasynaptic without ultrastructural or super-resolution analyses (Kater et al. 2023).

Second, GluA2 was used as a marker of GluA2-containing AMPAR-associated puncta, but it does not label all AMPAR populations. Therefore, GluA2-positive puncta should not be interpreted as equivalent to total AMPAR distribution.

Third, the present study did not include cell-type-specific markers in the same quantitative analysis. Consequently, the cellular origin of individual GluA2-positive and NR1-positive puncta cannot be determined. Given the cellular heterogeneity of the preBötC, which contains intermingled glutamatergic, inhibitory, and glial populations (Grass et al. 2004; Oke et al. 2023; Connelly et al. 2025), some puncta may arise from neuronal compartments, whereas others may originate from glial cells (Krasnow and Attwell 2016; Verkhratsky and Chvátal 2020; Picañol et al. 2026; Ahmadpour et al. 2024) or intracellular receptor pools. Thus, a subset of the puncta identified here, particularly those classified as non-PSD95-associated, may correspond to non-neuronal elements or intracellular receptor populations. Furthermore, NR1 immunoreactivity alone does not establish receptor surface expression, assembly into functional NMDAR complexes, association with specific GluN2 subunits, or physiological receptor activity.

Fourth, puncta identification relied on observer-guided threshold selection combined with predefined area and circularity criteria. Although all images were processed using identical inclusion criteria, puncta should be regarded as operationally defined immunoreactive objects rather than direct measures of receptor number or molecular organization.

Fifth, the GluA2–EAAT2 analysis provides an object-based measure of proximity between GluA2-positive puncta and EAAT2-positive domains. This analysis does not demonstrate physical contact, transporter activity, or functional coupling. Likewise, proximity thresholds used in the present study should be interpreted as operational measures of submicron spatial association rather than evidence of direct membrane apposition or tripartite synaptic organization. Although the enrichment of GluA2-positive puncta near EAAT2-positive domains remained robust across a range of thresholds (100–500 nm), the spatial resolution of confocal microscopy does not permit reliable determination of nanoscale astrocyte–synapse relationships. The uniform distance comparison used here should be considered exploratory because nearest-neighbor distance distributions are influenced by tissue geometry, object density, and EAAT2 domain occupancy.

Sixth, the present study is descriptive and anatomical in nature. The experiments performed here do not directly assess receptor current, glutamate dynamics, transporter activity, neuronal excitability, or respiratory network output. Accordingly, conclusions should be restricted to anatomical organization and spatial relationships among immunoreactive structures. Whether these anatomical arrangements vary with, or contribute to, changes in respiratory activity remains unknown and will require studies combining anatomical analyses with physiological measurements and experimental conditions that modify respiratory network function.

Finally, all analyses were performed in adult male rats. Because glutamate receptor expression and respiratory network properties change during development, the present findings should not be generalized directly to neonatal preparations.

Despite these limitations, the present study provides a quantitative anatomical description of glutamatergic signaling elements in the adult rat preBötC. The identification of a prominent non-PSD95-associated NR1-positive population and the close spatial association between GluA2-positive puncta and EAAT2-positive domains establish a framework for future studies addressing the cellular and ultrastructural organization of glutamatergic signaling within respiratory networks.

## Data Availability

The datasets generated and analyzed during the current study, including raw confocal image files, processed image-analysis outputs, and statistical analysis scripts, are available from the corresponding authors upon reasonable request.
